# Shared decision making, aggression, and coercion in inpatients with schizophrenia

**DOI:** 10.1192/j.eurpsy.2020.88

**Published:** 2020-09-28

**Authors:** Johannes Hamann, Miriam John, Fabian Holzhüter, Spyridon Siafis, Peter Brieger, Stephan Heres

**Affiliations:** 1 Klinik und Poliklinik für Psychiatrie und Psychotherapie, Klinikum rechts der Isar, Technische Universität München, München, Germany; 2 kbo Isar-Amper-Klinikum München Ost, Haar, Germany

**Keywords:** Autonomy, coercion, schizophrenia, shared decision making, violence

## Abstract

**Background:**

The present study aimed at answering three research questions: (a) Does shared decision making (SDM) yield similar effects for patients with involuntary admission or incidents of aggression compared to patients with voluntary admission or without incidents of aggression? (b) Does SDM reduce the number of patients with incidents of aggression and the use of coercive measures? (c) Does the use of coercion have a negative impact on patients’ perceived involvement in decision making?

**Methods:**

We used data from the cluster-randomized SDM-PLUS trial in which patients with schizophrenia or schizoaffective disorder in 12 acute psychiatric wards of 4 German psychiatric hospitals either received an SDM-intervention or treatment as usual. In addition, data on aggression and coercive measures were retrospectively obtained from patients’ records.

**Results:**

The analysis included *n* = 305 inpatients. Patient aggression as well as coercive measures mostly took place in the first days of the inpatient stay and were seldom during the study phase of the SDM-PLUS trial.

Patients who had been admitted involuntarily or showed incidents of aggression profited similarly from the intervention with regard to perceived involvement, adherence, and treatment satisfaction compared to patients admitted voluntarily or without incidents of aggression. The intervention showed no effect on patient aggression and coercive measures. Having previously experienced coercive measures did not predict patients’ rating of perceived involvement.

**Conclusion:**

Further research should focus on SDM-interventions taking place in the very first days of inpatients treatment and potential beneficial long effects of participatory approaches that may not be measurable during the current inpatient stay.

## Introduction

The model of shared decision making (SDM) aims to strengthen patients’ autonomy and encourage a more equal relationship between patients and doctors [1]. This fits well with the desire for a more ethical psychiatry [2]. The practicability of SDM in psychiatry and also in schizophrenia treatment has been shown in several trials [3], and there is even some evidence that SDM might be an option for patients on acute wards or those being admitted involuntarily [4,5]. Moreover, Stovell et al. [6] concluded that “…applying a shared decision-making approach to decisions about future treatment may reduce … the risk of patients experiencing compulsory care,” possibly indicating that SDM and (reduced) coercion might be linked. However, to our best knowledge, this potential link has not yet been studied.

The use of coercive measures, often a consequence of patient aggression, still constitutes a major problem and controversial issue in psychiatric care [7–9]. Both phenomena, aggression and coercion, are often linked and somehow conflicting with SDM. Applying force is the opposite of SDM, which promotes autonomy and patient rights [10]. Force or coercion may therefore have a negative impact on patients’ perceived involvement. Aggression (from the patients’ side) may be an indicator that provider–patient communication has failed. Being aggressive could therefore in some cases being judged as a sign of frustration that one’s voice had not been heard. An increased opportunity for patients to be heard might the other way round reduce aggression.

In our view, there are at least three research questions about potential relationships among SDM, patient aggression, and coercion that deserve further study:

Research question 1: Does SDM yield similar effects for patients with involuntary admission or incidents of aggression compared to patients with voluntary admission or without incidents of aggression?

Research question 2: Does SDM reduce the number of incidents of aggression and the use of coercive measures (potentially mediated by higher satisfaction with treatment and better adherence)?

Research question 3: Is there is a reciprocal relationship between SDM and coercion, that is does the use of coercion have a negative impact on patients’ perceived involvement in decision making?

Recently, the SDM-PLUS trial for acute inpatients with schizophrenia had been conducted including a considerable number of patients admitted involuntarily or having incidents of aggression [5]. In this cluster-randomized trial, an SDM-intervention, which addressed both inpatients with schizophrenia as well as their clinicians, was compared to treatment as usual. The analysis of the primary outcome for the whole sample has shown that the intervention improved patients’ perceived involvement in decision making and also their treatment satisfaction and therapeutic alliance [5].

Within the present post-hoc analysis, we aimed at answering the above cited research questions by using data from the SDM-PLUS trial.

## Methods

### Patient recruitment, randomization, and intervention in the initial SDM-PLUS trial

Data were gathered during the SDM-PLUS trial on 12 acute psychiatric wards of 4 psychiatric hospitals in Germany. Pairs of comparable wards (number of patients, distribution of diagnosis, staff, etc.) were determined. One ward of each pair was randomized to intervention and one to the control group (treatment as usual). Inclusion criteria were inpatient status of participating ward, age 18–65 years, diagnosis of schizophrenia or schizoaffective disorder (ICD 10: F20/F25), being capable of participating in 60 min, group intervention (according to their clinicians’ estimate), and being able to provide written informed consent. The only exclusion criteria were mental retardation and insufficient proficiency in German to discuss treatment decisions.

In the intervention group, a complex intervention was implemented, consisting of a staff training and a patient training in SDM PLUS. The SDM-PLUS approach aims to empower health care staff and patients alike with regard to SDM-specific communication techniques. For health care staff, the existing approaches to applying SDM were expanded to include patients without insight or with reduced decisional capacity. Therefore, SDM-PLUS teaches communication techniques derived from motivational interviewing and negotiation approaches [11].

Patients were provided with group training in SDM [12] and the use of question prompt sheets for ward rounds and individual consultations. Throughout the study period, this group training was offered twice a week for all wards and it was ensured that all intervention group patients participated at least in two group sessions. Staff (and patients) from the control wards acted under TAU conditions but were offered SDM-PLUS training after the end of the study.

In the primary analysis, the intervention has been shown to improve patients’ perceived involvement in decision making, their satisfaction with treatment, and their rating of the therapeutic alliance [5].

The present analysis is a post hoc analysis for which data on aggression and coercion were, after having received a review board approval for the respective amendment, retrospectively, collected for all patients having participated in the SDM-PLUS trial.

### Data on patient aggression and coercive measures

Data on incidents of patient aggression and coercive measures were retrospectively obtained from patient records of participants of the SDM-PLUS trial, using a specifically adapted scoring sheet. The researcher, who obtained data, was thereby blinded with regard to group allocation. Due to the design of the original trial, three time slots of individual duration were defined:

1. preintervention phase: hospital admission until study entry;
2. intervention phase: study entry until 3 weeks later or discharge (whatever happened first);
3. postintervention phase: end of intervention phase until discharge (in case patients were not discharged during intervention phase).

In the initial trial, the primary outcome (perceived involvement in decision making) was obtained at the end of the (3-week) intervention phase. For all three time slots, the respective number of days and all record notes regarding incidents of patient aggression and coercive measures were obtained from the records. To operationalize patient aggression, we used the modified overt aggression scale (MOAS), a validated rating tool to assess the alignment and severity of aggressive acts [13]. The MOAS originally subdivides incidents of aggression into the four different categories, verbal aggression, aggression against objects, auto aggression, and aggression against others, and allows a sum score to be formed. For the present analysis, we dichotomized MOAS sum scores (score of 0 = no aggression, any other score = aggression). This was done for all time slots separately. Incidents of coercion were also dichotomized (score of 0 = no mechanical restraint/forced medication, score of 1 = one or more incident of mechanical restraint/forced medication). Data on physical restraints and forced medication were available from patient records as it was mandatory in the participating hospitals to document these events separately.

Further data were obtained regarding legal guardianship (y/n), legal grounds at admission (involuntary/voluntary), and how many days after admission patients were allowed to leave the ward unattended for the first time.

To assess interrater reliability, two independent raters evaluated *N* = 10 patient records simultaneously and compared their ratings of single incidents (e.g., forced medication: yes/no; incidence of verbal aggression: yes/no). Overall there was an agreement in >90% of the ratings. For any discrepancies between the two raters specific definitions were formulated.

### Additional data

The data above were then merged with the original dataset of the SDM-PLUS trial. From this dataset, the following measures, previously shown to be related to aggression, nonadherence, or coercion, were used for the analyses: At study entry clinicians provided Clinical Global Impression (CGI) scores and patients filled in the Autonomy Preference Index [14], the MacArthur Admission Experience Survey [15], and the Birchwood Insight Scale [16].

Patients perceived involvement in decision making (SDM-Q-9 questionnaire) [17], their treatment satisfaction (ZUF 8 [18]), and their self-reported adherence (Medication Adherence Rating Scale [MARS] [19]) were obtained at the end of the intervention phase.

#### Statistical analysis

All analyses were of exploratory nature (post hoc analysis of observed cases) and were performed with SPSS and R. First, descriptive statistics were applied to describe the study population and the frequency of aggression and coercion, separately for the intervention and control group.

To test whether treatments effect on perceived involvement, self-reported adherence, and treatment satisfaction vary according to legal grounds on admission or incidents of aggression before study inclusion (research question 1), we fitted mixed-effects linear regression models similarly to the primary analysis [5], and added interaction terms for legal grounds at admission or incidents of aggression before study inclusion. Ward (cluster) was added as a random-effects term and intervention group as well as aggression or legal grounds at admission as fixed-effects term.

For testing the potential effect of the SDM-PLUS intervention on patient aggression and coercive measures during the intervention phase (research question 2), mixed-effects regression models were used with cluster as a random-effect term and intervention group as a fixed-effect. Thereby, we used linear regression models for continuous outcomes and logistic regression models for dichotomous outcomes. As there were statistically significant differences at baseline regarding involuntary admission and a higher proportion of patients showing incidents of aggression in the preintervention phase in the intervention group, we controlled for these two variables, by including both as fixed-effect factors in the models. The same analysis was repeated for the postintervention phase to study potential long-term effects of the intervention.

Finally, we used a linear regression model to study the influence of coercive measures on patients’ perceived involvement in decision making (SDM-Q-9) (research question 3). We included the following factors as independent variables: group (intervention/control), age, gender, legal grounds on admission (voluntary/ involuntary), previous number of inpatient stays, severity of illness at study entry (CGI-score), legal guardianship (y/n), Insight Scale sum score, MacArthur Admission Questionnaire sum score, Autonomy Preference Index sum score, and, in addition, incidents of coercive measures (restraint y/n, forced medication y/n) for the preintervention and intervention phases added (i.e., before obtaining the SDM-Q-9). For all analyses two-sided alpha was set at 0.05.

#### Ethics, informed consent procedure, and trial registration

The trial as well as the amendment regarding further data acquisition were approved by the local review board of the TU Munich (*Ethikkommission der Technischen Universität München*). All patients gave written informed consent. The trial was registered at *Deutsches Register Klinischer Studien* (DRKS00010880) and the initial study protocol has been published [20].

## Results

### Sample characteristics

*N* = 322 patients were recruited for the SDM-PLUS trial. For *N* = 305 patients, data on patient aggression and coercive measures could be obtained and matched to the original dataset, so this group constitutes the sample for the present analysis. Female and male patients were nearly equally frequent and the mean age was 42 years (median 43 years). Most patients were suffering from chronic courses of schizophrenia with a mean duration of illness of 12.4 years and a mean of 7 inpatient stays (see Table 1). There were no significant differences regarding baseline measures between the intervention and control group, apart from legal grounds at admission with more patients in the intervention group being admitted involuntarily.

**Table 1.** Socio-demographics and clinical data at baseline and aggressive incidents, number of mechanical restraints, and forced medication during preintervention phase.

	Intervention (*n* = 154)	Control (*n* = 151)	Group comparison
Age	42.4 (12.9)	41.2 (13.3)	*p* = 0.45
Gender (female, %)	82 (53%)	73 (48%)	*p* = 0.39
Diagnosis	F20: 95 (62%)	F20: 111 (74%)	*p* = 0.09
	F25: 49 (32%)	F25: 33 (22%)	
	Other F2: 10 (6%)	Other F2: 7 (5%)	
CGI at study entry	5.3 (0.9)	5.5 (0.8)	*p* = 0.16
Duration of illness	12.3 (10.3)	12.5 (10.8)	*p* = 0.88
Number of inpatient stays	7.0 (6.8)	7.5 (7.5)	*p* = 0.55
Patients admitted involuntarily	61 (40%)	42 (27%)	*p* = 0.03
Number of patients with at least one aggressive incident	52 (34%)	43 (28%)	*p* = 0.32
Verbal aggression	41	31	*p* = 0.22
Aggression against objects	20	17	*p* = 0.66
Aggression against persons	24	18	*p* = 0.37
Auto aggression	6	4	*p* = 0.55
Number of patients exposed to mechanical restraints	35 (23%)	31 (21%)	*p* = 0.64
Number of patients receiving involuntary drug treatment	17 (11%)	12 (8%)	*p* = 0.36
Mean length of preintervention phase (days)	21.7 (31.3)	18.0 (25.9)	*p* = 0.26

*Note:* Values in parentheses are standard deviations for metrical variables and percentages for frequency variables. Metrical variables were tested by means of the *t*-test for independent samples. Frequency variables were tested by means of the *χ* ^2^-test.

Abbreviation: CGI, clinical global impression.

Overall, 103 patients had been admitted involuntarily (32%). For 111 patients, at least one aggressive incident had been recorded over the whole inpatient stay. Seventy-six patients had been physically restrained at least once during the inpatient stay and 31 patients were given medication on an involuntary basis.

**Figure 1:**
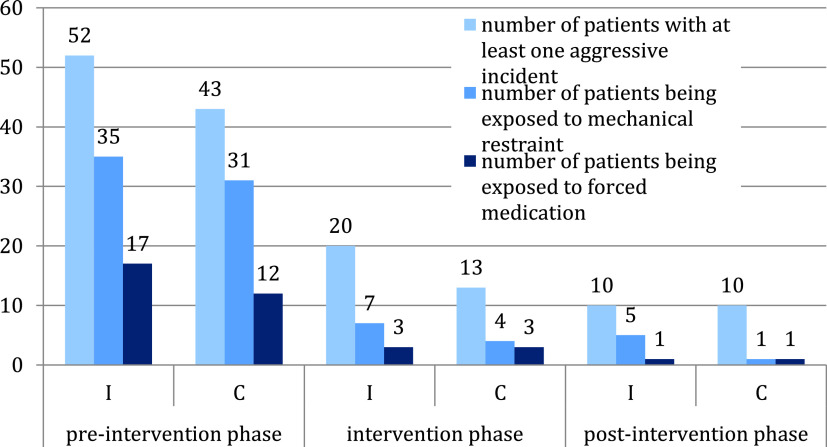
Incidence of patient aggression and measures of coercion.

As displayed in Figure 1, most aggressive incidents and most acts of coercion occurred during the first days of inpatient treatment and before the SDM-PLUS study started.

### Intervention effects on perceived involvement in decision making by subgroup

The number of participants with data available for this analysis was 242; 122 participants in the intervention group (50 with involuntary admission and 42 with aggression before study inclusion) and 120 in the control group (36 with involuntary admission and 35 with aggression before study inclusion). No subgroup differences were found, except for a larger mean difference in SDM-Q-9-scores for participants with involuntary admission compared to voluntary, yet the *p*-value was marginally significant (26.1 vs. 12.4; *t*-test = 1.988, *df* = 228, *p* = 0.048; Table 2). Therefore, subgroup analyses indicated that relative effects of SDM-PLUS intervention on perceived involvement in decision making might not differ between subgroups, or they could be even larger, for patients with involuntary admission or aggressive incidents.

**Table 2.** Intervention effects on perceived involvement in decision making by subgroup.

Outcome	Group	*n*	Mean difference of intervention versus control	Test for interaction
Perceived involvement in decision making (SDM-Q-9)	Involuntary admission	*I* = 50, *C* = 36	26.1, 95% CI (13.7, 38.5), SE = 5.6, *p* < 0.001	*t*-test = 2.0, *p* = 0.048
	Voluntary admission	*I* = 72, *C* = 84	12.4, 95% CI (3.3, 21.5), SE = 4.1, *p* = 0.013	
Self-reported adherence (MARS)	Involuntary admission	*I* = 50, *C* = 36	1.8, 95% CI (0.5, 3), SE = 0.6, *p* = 0.011	*t*-test = 1.0, *p* = 0.32
	Voluntary admission	*I* = 72, *C* = 83	1.1, 95% CI (0.1, 2.1), SE = 0.4, *p* = 0.031	
Treatment satisfaction (ZUF8)	Involuntary admission	*I* = 50, *C* = 36	4.3, 95% CI (1.4, 7.2), SE = 1.3, *p* = 0.007	*t*-test = 1.1, *p* = 0.26
	Voluntary admission	*I* = 72, *C* = 84	2.6, 95% CI (0.4, 4.8), SE = 1, *p* = 0.024	
Perceived involvement in decision making (SDM-Q-9)	Aggression at baseline	*I* = 42, *C* = 36	23.2, 95% CI (9.6, 36.9), SE = 6.1, *p* = 0.003	*t*-test = 1.4, *p* = 0.16
	No aggression at baseline	*I* = 80, *C* = 84	13.1, 95% CI (3.5, 22.8), SE = 4.3, *p* = 0.012	
Self-reported adherence (MARS)	Aggression at baseline	*I* = 42, *C* = 35	1.2, 95% CI (−0.2, 2.6), SE = 0.6, *p* = 0.076	*t*-test = −0.1, *p* = 0.90
	No aggression at baseline	*I* = 80, *C* = 84	1.3, 95% CI (0.3, 2.3), SE = 0.4, *p* = 0.015	
Treatment satisfaction (ZUF8)	Aggression at baseline	*I* = 42, *C* = 36	3.2, 95% CI (0, 6.4), SE = 1.4, *p* = 0.052	*t*-test = 0.2, *p* = 0.81
	No aggression at baseline	*I* = 80, *C* = 84	2.8, 95% CI (0.4, 5.2), SE = 1.1, *p* = 0.027	

Abbreviations: *C*, control group; *I*, intervention group; MARS, medication adherence rating scale; SDM-Q, Shared Decision Making Questionnaire. CI: confidence interval SE: standard error.

### Effects of SDM on incidents of aggression and coercive measures

The SDM-PLUS intervention had no significant effects on patient aggression or on coercive measures such as mechanical restraint or forced medication during the intervention phase. There were also no significant effects in patients with continued inpatient treatment after the intervention phase regarding these incidents. Likewise, there were no group differences regarding days until first unattended leave from ward (Table 3).

**Table 3.** Aggressive incidents, number of mechanical restraints, and forced medication during intervention phase and postintervention phase.

	Intervention (*n* = 154)	Control (*n* = 151)	Univariate group comparison (*t*-test, *χ* ^2^ test)	Mixed effects regression models
Intervention phase				
Mean length of intervention phase	17.6 (5.7)	16.2 (6.5)	*p* = 0.045	MD = 1.23, 95% CI (−0.72, 3.18), SE = 0.88, *p* = 0.191
Number of patients with at least one aggressive incident	20 (13%)	13 (9%)	*p* = 0.22	OR = 1.58, 95% CI (0.63, 3.97), SE = 0.47, *p* = 0.335
Verbal aggression	17 (11%)	8 (5%)	*p* = 0.05	OR = 2.19, 95% CI (0.86, 5.57), SE = 0.48, *p* = 0.100
Aggression against objects	7 (5%)	7 (5%)	*p* = 0.96	OR = 0.94, 95% CI (0.31, 2.83), SE = 0.56, *p* = 0.916
Aggression against persons	8 (5%)	4 (3%)	*p* = 0.26	OR = 2.24, 95% CI (0.65, 7.77), SE = 0.63, *p* = 0.202
Auto aggression	0 (0%)	2 (1%)	*p* = 0.15	Not estimable
Number of patients being mechanically restrained at least once	7 (5%)	4 (3%)	*p* = 0.37	OR = 1.48, 95% CI (0.33, 6.64), SE = 0.77, *p* = 0.607
Number of patients receiving involuntary drug treatment	3 (2%)	3 (2%)	*p* = 0.98	OR = 0.38, 95% CI (0.03, 5.58), SE = 1.38, *p* = 0.478

	Intervention (*n* = 93)	Control (*n* = 77)	Univariate group comparison (*t*-test, *χ* ^2^ test)	Mixed effects regression models
Postintervention phase				
Mean length of post intervention phase	38.7 (41.2)	37.6 (45.2)	*p* = 0.87	MD = 1.61, 95% CI (−20.25, 23.48), SE = 9.81, *p* = 0.873
Number of patients with at least one aggressive incident	10 (11%)	10 (13%)	*p* = 0.65	OR = 0.76, 95% CI (0.27, 2.15), SE = 0.53, *p* = 0.601
Verbal aggression	9 (10%)	9 (12%)	*p* = 0.67	OR = 0.81, 95% CI (0.25, 2.65), SE = 0.60, *p* = 0.734
Aggression against objects	2 (2%)	0 (0%)	*p* = 0.20	Not estimable
Aggression against persons	4 (4%)	2 (3%)	*p* = 0.55	OR = 1.56, 95% CI (0.26, 9.28), SE = 0.91, *p* = 0.624
Auto aggression	0 (0%)	1 (1%)	*p* = 0.27	Not estimable
Number of patients being mechanically restrained at least once	5 (5%)	1 (1%)	*p* = 0.15	OR = 3.61, 95% CI (0.40, 32.75), SE = 1.13, *p* = 0.254
Number of patients receiving involuntary drug treatment	1 (1%)	1 (1%)	*p* = 0.89	OR = 0.24, 95% CI (0.24, 3996.6), SE = 4.97, *p* = 0.771
Days until first unaccompanied leave from ward	20.3 (22.9)	19.0 (24.5)	*p* = 0.64	MD = −1.22, 95% CI (−13.83, 11.39), SE = 5.66, *p* = 0.833

Abbreviations: CI: confidence interval MD: mean difference OR: Odds ratio SE: standard error.

### Relationship between coercion and SDM

Patients’ perceived involvement in decision making (obtained at the end of the intervention phase) was predicted by group allocation (in favor of the intervention group *B* = −17.9, *p* < 0.001), younger age (*B* = −0.26, *p* = 0.05), more insight (Insight Scale, *B* = 1.28, *p* = 0.04) and lower participation preferences (Autonomy Preference Index, *B* = −1.41, *p* = 0.002), but not by incidents of mechanical restraints (*B* = 1.91, *p* = 0.69) or forced medication (*B* = −8.77, *p* = 0.16) during the preintervention and intervention phases. The regression model predicted 28% of the variance (*R* ^2^ = 0.28).

## Discussion

The relative treatment effects of the SDM-PLUS intervention did not differ with regard to patients’ perceived involvement in decision making for patients with involuntary (vs. voluntary admission) and for patients with incidents of aggression (vs. no aggression). The intervention showed no effect on patient aggression and the number patients being exposed to coercive measures. In addition, having experienced coercive measures did not predict patients’ rating of perceived involvement. For the whole sample, patient aggression as well as coercive measures mostly took place in the first days of the inpatients’ stay and before patient inclusion in the SDM-PLUS trial.

### Limitations

We present results of a post-hoc analysis. The initial trial was not designed nor powered to measure the influence of SDM-PLUS on patient aggression. Incidents of aggression and the use of coercive measures were retrospectively obtained from patient records, making it likely that these incidents were underestimated compared to a study design in which patient aggression is documented prospectively and therefore awareness regarding such events raised. In addition, most events of aggression and coercion took place in the first days of the inpatient stay and thereby before study inclusion of patients. While this finding is in line with previous analyses [21], this may have reduced the validity of our analysis. Statistically significant findings should also be interpreted with caution, since adjustment to multiple testing was not conducted, given the exploratory nature of our analysis. Furthermore, it is challenging to oversee the full extent of how hospital staff actually implemented the communication training on wards and performed it during decision making. Finally, we included only patients who were capable of attending 60 min of group session. This procedure may have led to an overrepresentation of patients with rather good remission or who gained insight during the course of inpatient treatment.

### Interpretation of results

Our subgroup analyses indicated that SDM might not differ in a group of patients in which doctor–patient decision making is often considered difficult [22], namely inpatients being admitted involuntarily or those showing incidents of aggression. This is an important result, giving the skepticism towards SDM in this population. However, the intervention did not lead to fewer incidents of patient aggression or to a lower use of coercive measures. The idea behind this second research question was that patients participating in decision making to a greater extent might be less likely to be frustrated or aggressive. Likewise, clinicians trained in sharing decisions and motivational interviewing might be less prone to use coercive measures. However, the hypothesized effect was not shown in our analysis. We believe that mainly methodical reasons account for this finding. Thus, most incidents of aggression took place in the preintervention phase and therefore before the intervention. Consequently, the number of aggressive or coercive events during the intervention phase was very low. Thus, the intervention might just have come too late or we might not have been able to show a correlation, even if it existed, due to the low number of incidents during the intervention phase.

The third research question was whether the experience of coercion reduces patients’ perceived involvement in decision making. In other words, we wanted to find out whether applying coercive measures as a clinician destroys any opportunity to switch to SDM in later stages of inpatient treatment. This idea would be supported by patients’ statements that the experience of powerlessness (e.g., coercive measures) leads to less participation in decision making during later treatment [23] or by findings that perceived coercion deteriorates therapeutic alliance [24], yet it was not supported by our results. Thus, patients having experienced coercion in the first days of inpatient treatment did not feel less involved in decision making at later stages, indicating that it is never too late to start with SDM, even if it was not possible during admission.

## Conclusion

As the SDM-intervention showed benefits regarding perceived involvement, adherence and treatment satisfaction but not regarding aggressive behavior or the use of coercive measures further research should focus on two issues raised by our study: (a) SDM-interventions taking place in the very first days of inpatient treatment and (b) potential beneficial long-term effects of participatory approaches [6, 25] that may not be measurable during the present inpatient stay.

## Data Availability

The datasets generated during and/or analyzed during the current study are available from the corresponding author on reasonable request.
